# Sustainable return to work among breast cancer survivors

**DOI:** 10.1002/cam4.6467

**Published:** 2023-08-21

**Authors:** Garazi Ruiz de Azua, Isabelle Kousignian, Ines Vaz‐Luis, Antonio Di Meglio, Elsa Caumette, Julie Havas, Elise Martin, Anne‐Laure Martin, Ophelie Querel, Laurence Vanlemmens, Barbara Pistilli, Charles Coutant, Paul Henri Cottu, Asma Dhaini Merimeche, Florence Lerebours, Olivier Tredan, Christelle Jouannaud, Christelle Levy, Agnes Dumas, Gwenn Menvielle

**Affiliations:** ^1^ Sorbonne Université, INSERM, Institut Pierre Louis d'Épidémiologie et de Santé Publique, IPLESP, Équipe de Recherche en Épidémiologie Sociale Paris France; ^2^ Université Paris Cité, Unité de Recherche «Biostatistique, Traitement et Modélisation des données bio‐logiques» BioSTM, UR 7537 Paris France; ^3^ Medical Oncology Department Gustave Roussy Villejuif France; ^4^ INSERM Unit 981—Prédicteurs moléculaires et nouvelles cibles en oncologie, Gustave Roussy Villejuif France; ^5^ Department of Maieutics Université de Montpellier Montpellier France; ^6^ UCBG, UNICANCER Paris France; ^7^ Centre Oscar Lambret Lille France; ^8^ Breast Cancer Group Institut Gustave‐Roussy Villejuif France; ^9^ Department of Medical Oncology Centre Georges‐François Leclerc Dijon France; ^10^ Department of Medical Oncology Institut Curie Paris France; ^11^ Centre Alexis Vautrin Vandoeuvre Les Nancy France; ^12^ Department of Medical Oncology Institut Curie Saint‐Cloud France; ^13^ Centre Léon Berard Lyon France; ^14^ Institut Jean Godinot Reims Reims France; ^15^ Centre François Baclesse Caen France; ^16^ Université Paris Cité ECEVE, UMR 1123, Inserm Paris France

**Keywords:** breast cancer, cohort, employment, epidemiology, quality of life

## Abstract

**Purpose:**

This study assessed sustainable return to work (SRTW) of breast cancer survivors (BCS).

**Methods:**

We used data from the prospective French cohort, CANTO. We included 1811 stage I–III BCS who were <57 years old and employed at the moment of diagnosis and working 2 years after diagnosis. Using logistic regression, we investigated the role of clinical, health and socio‐economic factors, and the work environment on SRTW 3 years after diagnosis. We compared having any sick leave with having worked continuously and being unemployed to having worked continuously between 2 and 3 years after diagnosis.

**Results:**

Overall, 77% (*n* = 1395) worked continuously after return to work (RTW). Out of the other 416 BCS, 66% had any sick leave period, 33% had been unemployed, 4% had an early retirement, 2% a disability and 1% another status (multiple situations possible). Being on sick leave was associated with age > 50 (OR = 0.59; 95%CI = 0.43–0.82), stage III (2.56; 1.70–3.85), tumour subtype HR+/HER2+ (0.61; 0.39–0.95), severe fatigue (1.45; 1.06–1.98), workplace accommodations (1.63; 1.14–2.33) and life priorities (0.71; 0.53–0.95). Unemployment was associated with age > 50 (0.45; 0.29–0.72), working in the public sector (0.31; 0.19–0.51), for a small company (3.00; 1.74–5.20) and having a fixed‐term contract (7.50; 4.74–11.86).

**Conclusions:**

A high number of BCS have periods of sick leave or unemployment after RTW. The determinants differ between sick leave and unemployment.

**Implications for cancer survivors:**

BCS need to be supported even after RTW, which should be regarded as a process.

## 1 INTRODUCTION

Breast cancer (BC) is the most common cancer irrespective of sex.^1^ Annually around 2.26 million women are diagnosed with BC worldwide.^1^ In high income countries, one third of them are younger than 55 years old at the moment of diagnosis,^1^ implying they are still at a working age. With 5‐year survival rates reaching 85% in many high income countries,^1^ employment after cancer is becoming a major challenge for BC survivors (BCS) and society, from a public health and an economic view. From the public administration perspective, great costs are associated to expenses other than treatment. For example, in Sweden, 70% of costs linked to all cancer expenses are not treatment related.^2^ From survivors' perspective, a cancer diagnosis can have a considerable impact on their professional life and financial status,^3^ and work life is an important part of the recovery process, giving a sense of normality and of being productive within society.^4^ A recent meta‐analysis found that 70% of BCS return to work after the end of treatment, which was not significantly different from all cancer survivors.^5^ However, the challenge goes beyond return to work (RTW) and sustainable RTW (SRTW) is an issue for many BCS who often have several sick leaves or struggle to retain their jobs after RTW.^6^, ^7^, ^8^, ^9^, ^10^

Employment after cancer can be affected by different factors. Evidence from studies including survivors from all cancer sites found that clinical factors, sociodemographic factors, including older age, low socio‐economic status, workplace environment (e.g. discrimination) and work‐related factors (e.g. type of contract), as well as the implementation of workplace accommodations are important factors associated with job retention and sick leave.^11^, ^12^, ^13^, ^14^, ^15^, ^16^, ^17^, ^18^ Among BCS, the literature is scarce. Women receiving chemotherapy, with treatment‐related sequelae (e.g. pain and fatigue) and poor mental health are more likely to have prolonged sick leave or early retirement.^6^, ^7^, ^8^, ^9^ Regarding the association between changes in work status and sociodemographic factors, some studies have suggested household composition is associated.^7^, ^8^ None of these studies that included only BCS investigated administrative working factors in their analyses.

However, most studies on job trajectories after BC have focused on global trajectories, from diagnosis to exit from employment several years after diagnosis.^6^, ^7^, ^8^, ^9^, ^11^, ^16^ These studies do not differentiate between survivors who never returned to work and those who did and then leave their positions. This complicates the distinction between factors associated with RTW from those associated with SRTW. However, understanding the characteristics associated with not working after having returned to work is essential to help BCS in their RTW process. Therefore, we deem it essential to study the sub‐population of survivors who returned to work after treatment, and the factors associated with consecutive sick leave or exit from employment.

In view of this gap, our study aimed at studying SRTW of BCS who had returned to work after treatment. This study has two objectives: (1) to estimate SRTW 3 years after diagnosis among BCS that returned to work after treatment and (2) to study the effect of clinical, health‐related, sociodemographic and work‐related factors on SRTW.

## 2 METHODS

### 2.1 Study design

**FIGURE 1:**
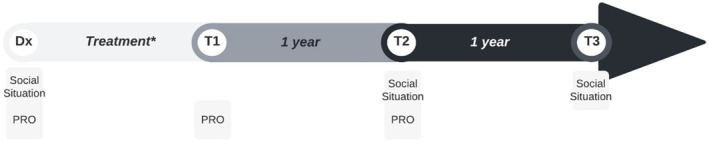
Timeline of data collection for the CANTO cohort. Dx, diagnosis; PRO, Patient‐reported outcome. T1, T2 and T3 correspond to approximately 1, 2 and 3 years after diagnosis. *Treatment lasted 6–9 months and T1 took place 3–6 months after treatment was completed.

Data were obtained from the CANcer TOxicities (CANTO) cohort. This is a French prospective cohort that includes women diagnosed with BC stage I–III. Cohort participants were recruited at the moment of diagnosis across Metropolitan France between 2012 and 2018.^19^ As shown in Figure 1, data were collected at diagnosis, and 1, 2 and 3 years after diagnosis (T1, T2 and T3 respectively) through self‐reported questionnaires, patient‐reported outcomes and during a clinical exam.

### 2.2 Study population

**FIGURE 2:**
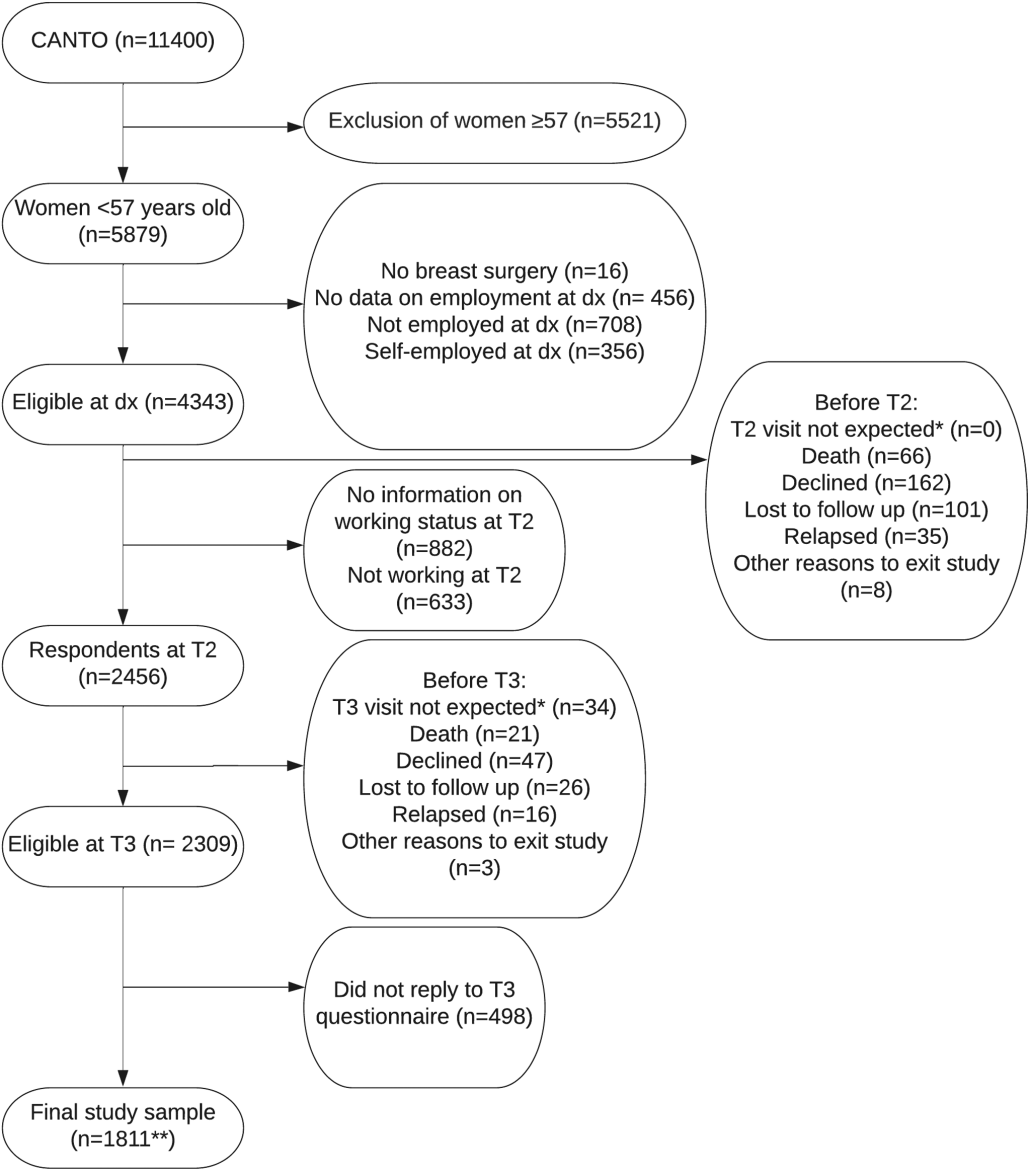
Flow chart of the study population from CANTO cohort participants. *Visit not expected: <1 year has elapsed since last visit. BC, breast cancer; dx, diagnosis; T1, T2 and T3, 1, 2 and 3 years post‐diagnosis. **1671 had information on whether they had any sick leave between T2 and T3, and 1530 had information on whether they had any period of unemployment between T2 and T3.

The CANTO cohort is comprised of 11,400 women. For the purpose of this study, we included women <57 years old (*n* = 5879) (to allow a 5‐year elapse before they reach the minimum retirement age), who had undergone surgery and were employed at the moment of diagnosis (self‐employed were excluded) (*n* = 4343), who were working at T2 (*n* = 2456) and provided information on their working status in the T3 questionnaire (*n* = 1811) (Figure 2). Due to the absence of information at T1 to determine working status (refer to Figure 1), we specifically chose to include only BCS who were working at T2.

### 2.3 Outcome

The main outcome of this study will be SRTW measured at T3. For this, we created two outcomes. A first outcome was created as having any sick leave of at least 1 month (cumulative) between T2 and T3 versus having worked continuously between T2 and T3 (reference category). Then, a second outcome was categorised as being unemployed at any moment between T2 and T3 versus having worked continuously between T2 and T3 (reference category).

### 2.4 Covariates

Covariates included in the model involved sociodemographic, clinical, health‐related and work‐related factors that were identified based on the existing literature.

#### 2.4.1 Sociodemographic variables

Age at diagnosis (<50, ≥50), living with a partner at T2 (No/Yes), having economically dependent children at T2 (No/Yes) and total household income at T2 (<2500€, 2500–5000€, ≥5000€).

#### 2.4.2 Clinical variables

Stage at diagnosis (I, II and III), tumour subtype at diagnosis (HR−/HER2−; HR+/HER2+; HR−/HER2+; HR+/HER2−).

#### 2.4.3 Health status at T2

Level of fatigue and physical functioning were assessed by the European Organisation for Research and Treatment of Cancer (EORTC) Quality of Life Questionnaire [QLQ]‐C30 and measured using the fatigue and physical functioning subscales (0–100), where an increase in the scale implies an increase in fatigue and physical functioning levels respectively. A cut off of 40 points was used to define severe fatigue (Yes/No), while 83 was used to define physical functioning (Poor/Good).^20^ Distress was evaluated using the general Hospital Anxiety and Depression Scale (HADS), which combines the anxiety and depression specific subscales: 0–12: normal, 13–18: borderline and 19–42: case.^21^

#### 2.4.4 Work‐related variables at T2

Having a permanent contract (Yes/No), size of company (<50 employees, 50–250 employees and >250 employees), working in the public sector (Yes/No), having a part−/full‐time contract, considering professional life more than or as important as personal life (Yes/No), having workplace accommodations (No/Yes) and having self‐reported perceived discrimination from employer after RTW (i.e. penalised because of cancer by their employer) (No/Yes). Cohort participants were asked if they had returned to work because of fear of losing their job if they did not (No/Yes).

### 2.5 Statistical analyses

The associations between sociodemographic, clinical, health and work‐related factors and the outcomes were studied using univariable and multivariable logistic regression models. All missing data on covariates were imputed using multiple imputations by fully conditional specification method with 30 imputations (the outcomes were not imputed).^22^ All analyses were performed using R version 4.0.5. Results will be expressed in terms of crude and adjusted odds ratios (OR, ORa) and their associated confidence intervals (95%CI). In addition, we performed a supplementary analysis (results are shown in Table S2) to study the association between the factors abovementioned and having any period of unemployment, an early retirement or having benefited of a disability status between T2 and T3 versus having worked continuously between T2 and T3. We considered BCS benefited from a disability status if they had declared having a recognised disability status, which in France is a status provided by the social security based on a medical assessment of the limitations to work.^23^

## 3 RESULTS

Most BCS, 1395 (77%), who were working 2 years after diagnosis continued to work in the next year without periods of sick leave, unemployment, retirement or disability. Out of the other 416 BCS, 276 (66%) had any sick leave, 135 (33%) had been unemployed between T2 and T3, 16 (4%) had an early retirement, 7 (2%) benefited from a disability status and 6 (1%) had an unspecified status. Some BCS had several events between T2 and T3; 22 BCS who had any sick leave period between T2 and T3 also had an unemployment period between T2 and T3. Out of the 276 BCS with any sick leave between T2 and T3, 179 (65%) were working at T3. Similarly, 85 (65%) of BCS who were unemployed between T2 and T3 were working at T3. The general characteristics of the study population after imputation have been summarised in Table 1 (see Table S1 for distribution before imputation). Overall, 61% of BCS were less than 50 years old at diagnosis, 90% had been diagnosed with stage I–II BC and had a good overall mental and physical health and 63% and 77% respectively. Most women in the study population had a permanent contract and worked for the public sector. Looking at the work environment, workplace accommodations were commonly put in place on their RTW (61%), 26% reported perceived discrimination and 19% had returned to work because they feared they would lose their job otherwise. When asked about their life priorities 43% of the study population responded their professional life was as or more important than their private life 2 years after diagnosis.

**TABLE 1:** Distribution of complete study sample and by having worked continuously, any sick leave or any unemployment period between T2 and T3. Imputed data set.

	All (100%)	Worked continuously (100%)	Any sick leave (100%)	Any unemployment (100%)
Sociodemographic characteristics at diagnosis				
Age ≥ 50 (ref. <50)	39	41	29	30
Clinical factors at diagnosis				
Stage at dx (ref. I)	47	48	37	51
II	43	43	43	44
III	10	8	19	5
Tumour subtype (ref. HR+/HER2−)	74	73	76	80
HR+/HER2+	12	13	10	12
HR−/HER2+	4	4	7	3
HR−/HER2−	10	10	7	6
Health status 2 years after diagnosis				
Good physical functioning (ref. Bad)	77	79	68	76
Severely fatigued (ref. No)	37	34	49	38
Distress (ref. No distress)	63	65	56	62
Distress: Borderline	25	25	25	24
Distress: Case	12	10	19	15
Sociodemographic characteristics 2 years after diagnosis				
Household income (ref. <2500€)	24	23	26	32
2500€–5000€	56	57	56	57
>5000€	19	20	18	11
Lives with partner (ref. No)	77	77	75	74
Has economically dependent children (ref. No)	64	64	66	60
Work and workplace factors 2 years after diagnosis				
Had workplace accommodations (ref. No)	61	60	79	45
Reported perceived discrimination (ref. No)	26	24	36	26
Size of company (ref. Large)	43	44	49	20
Medium	22	22	21	22
Small	35	33	30	59
Has a fixed‐term contract (ref. No)	14	12	12	43
Works part‐time (ref. No)	37	34	51	44
Works in the public sector (ref. No)	45	46	45	25
Returned to work because of fear of job loss (ref. No)	19	18	24	26
Professional life is as or more important than private life (ref. No)	43	45	34	44

Abbreviation: T2, T3, 2 and 3 years after diagnosis.

In the univariable analyses of having any sick leave versus having worked continuously between T2 and T3 (Table 2), we observed a positive association between having a sick leave between T2 and T3 and stage III, tumour subtype (HR−/HER2+), being severely fatigued, being distressed, having workplace accommodations, reporting discrimination from employers, working part‐time and returning to work because of fear of losing their job and a negative association with age, good physical functioning and prioritising professional over private life. The fully adjusted model showed an independent and positive association between having any sick leave between T2 and T3 and stage III, severe fatigue, having workplace accommodations and working part‐time and a negative association with age > 50, tumour subtype (HR+/HER2+) and prioritising professional over private life.

**TABLE 2:** Association between having any sick leave between T2 and T3 and sociodemographic, clinical, health and work‐related factors compared to working continuously between T2 and T3 (logistic regression) (*N* = 1671).

	Univariable models^a^		Multivariable models^a^	
	OR	[95%CI]	ORa	[95%CI]
Sociodemographic characteristics at dx				
Age > =50 (ref. <50)	0.61	[0.46;0.80]	0,59	[0.43;0.82]
Clinical factors at dx				
Stage at dx (ref. I)				
II	1.29	[0.97;1.72]	1.22	[0.90;1.65]
III	2.99	[2.03;4.40]	2.56	[1.70;3.85]
Tumour subtype (ref. HR+/HER2−)				
HR+/HER2+	0.78	[0.51;1.18]	0.61	[0.39;0.95]
HR−/HER2+	1.85	[1.05;3.24]	1.59	[0.88;2.88]
HR−/HER2−	0.71	[0.43;1.15]	0.62	[0.37;1.04]
Health status 2 years after dx				
Good physical functioning^b^ (ref. Bad)	0.57	[0.43;0.75]	0.87	[0.62;1.22]
Severely fatigued^b^ (ref. No)	1.86	[1.43;2.41]	1.45	[1.06;1.98]
Distress^b^ (ref. No distress)				
Distress: Borderline	1.17	[0.85;1.59]	0.88	[0.63;1.24]
Distress: Case	2.14	[1.49;3.07]	1.43	[0.95;2.17]
Sociodemographic characteristics 2 years after dx				
Household income (ref. <2500€)				
2500€–5000€	0.87	[0.64;1.19]	1.00	[0.67;1.49]
>5000€	0.79	[0.53;1.18]	1.03	[0.62;1.71]
Lives with partner (ref. No)	0.88	[0.65;1.19]	0.83	[0.55;1.24]
Has economically dependent children (ref. No)	1.09	[0.83;1.44]	0.85	[0.61;1.17]
Work and workplace factors 2 years after dx				
Had workplace accommodations (ref. No)	2.49	[1.82;3.42]	1.63	[1.14;2.33]
Reported perceived discrimination (ref. No)	1.79	[1.36;2.36]	1.09	[0.80;1.50]
Size of company (ref. Large)				
Medium	0.84	[0.59;1.19]	0.90	[0.62;1.30]
Small	0.79	[0.58;1.08]	0.85	[0.60;1.19]
Has a fixed‐term contract (ref. No)	0.98	[0.65;1.48]	1.07	[0.68;1.66]
Works part‐time (ref. No)	2.05	[1.57;2.67]	1.69	[1.26;2.26]
Works in the public sector (ref. No)	0.97	[0.74;1.26]	0.93	[0.69;1.24]
Returned to work because of fear of job loss (ref. No)	1.43	[1.05;1.96]	1.19	[0.84;1.68]
Professional life is as or more important than private life (ref. No)	0.62	[0.47;0.82]	0.71	[0.53;0.95]

*Note*: Distress was evaluated using the general subscale of HADS, which combines the anxiety and depression‐specific subscales of the Hospital Anxiety and Depression Scale (HADS). 0–12: normal, 13–18: borderline and 19–42: case.

Abbreviations: dx, diagnosis; OR, crude odds ratio; ORa, adjusted odds ratio; T2, T3, 2 and 3 years after diagnosis.

^a^ Reference category is having worked continuously between T2 and T3.

^b^ Severe fatigue and physical functioning were measured using the Quality of Life Questionnaire Core 30 Items (QLQC30) fatigue and physical functioning subscales (0–100). For fatigue, values >40 were considered as severely fatigued: For physical functioning, values >83 were considered as good physical functioning.

Table 3 shows the results of the univariable and multivariable analyses of being unemployed at any moment between T2 and T3 versus having worked continuously between T2 and T3. In the univariable analyses, we observe that being unemployed between T2 and T3 was positively associated with working for a medium or a small company, having a fixed‐term contract, working part‐time and returning to work because of fear of job loss. Meanwhile, being unemployed between T2 and T3 was negatively associated with age > 50, having a >5000€ household income, having workplace accommodations and working in the public sector. The fully adjusted model showed that being unemployed between T2 and T3 was positively and independently associated with working for a small company, having a fixed‐term contract and working part‐time and negatively and independently associated with age > 50, having workplace accommodations and working in the public sector.

**TABLE 3:** Association between having any period of unemployment at any moment between T2 and T3 and sociodemographic, clinical, health and work‐related factors compared to working continuously between T2 and T3 (logistic regression) (*N* = 1530).

	Univariable models^a^		Multivariable models^a^	
	OR	[95%CI]	ORa	[95%CI]
Sociodemographic characteristics at dx				
Age > =50 (ref. <50)	0.61	[0.42;0.90]	0.45	[0.29;0.72]
Clinical factors at dx				
Stage at dx (ref. I)				
II	0.95	[0.66;1.37]	0.96	[0.64;1.46]
III	0.59	[0.26;1.31]	0.67	[0.28;1.62]
Tumour subtype (ref. HR+/HER2−)				
HR+/HER2+	0.82	[0.47;1.43]	0.99	[0.53;1.84]
HR−/HER2+	0.68	[0.21;2.15]	0.87	[0.24;3.11]
HR−/HER2−	0.56	[0.27;1.18]	0.54	[0.24;1.20]
Health status 2 years after dx				
Good physical functioning^b^ (ref. Bad)	0.84	[0.55;1.28]	0.85	[0.50;1.44]
Severely fatigued^b^ (ref. No)	1.20	[0.83;1.72]	1.01	[0.64;1.59]
Distress^b^ (ref. No distress)				
Distress: Borderline	1.01	[0.66;1.56]	0.98	[0.60;1.61]
Distress: Case	1.50	[0.89;2.53]	1.33	[0.69;2.56]
Sociodemographic characteristics 2 years after dx				
Household income (ref. <2500€)				
2500€–5000€	0.72	[0.48;1.07]	1.08	[0.64;1.81]
>5000€	0.39	[0.21;0.73]	0.78	[0.36;1.69]
Lives with partner (ref. No)	0.83	[0.55;1.25]	0.72	[0.42;1.23]
Has economically dependent children (ref. No)	0.84	[0.58;1.22]	0.73	[0.46;1.15]
Work and workplace factors 2 years after dx				
Had workplace accommodations (ref. No)	0.56	[0.39;0.81]	0.58	[0.36;0.93]
Reported perceived discrimination (ref. No)	1.12	[0.74;1.70]	1.35	[0.81;2.25]
Size of company (ref. Large)				
Medium	2.17	[1.21;3.90]	1.82	[0.99;3.37]
Small	3.96	[2.44;6.43]	3.00	[1.74;5.20]
Has a fixed‐term contract (ref. No)	5.51	[3.73;8.13]	7.50	[4.74;11.86]
Works part‐time (ref. No)	1.55	[1.06;2.28]	1.64	[1.02;2.62]
Works in the public sector (ref. No)	0.40	[0.26;0.61]	0.31	[0.19;0.51]
Returned to work because of fear of job loss (ref. No)	1.63	[1.08;2.47]	1.39	[0.86;2.23]
Professional life is as or more important than private life (ref. No)	0.95	[0.66;1.38]	0.86	[0.57;1.31]

*Note*: Distress was evaluated using the general subscale of HADS. which combines the anxiety and depression‐specific subscales of the Hospital Anxiety and Depression Scale (HADS). 0–12: normal, 13–18: borderline and 19–42: case.

Abbreviations: dx, diagnosis; ORa, adjusted adds ratio; T2, T3, 2 and 3 years after diagnosis.

^a^ Reference category is having worked continuously between T2 and T3.

^b^ Severe fatigue and physical functioning were measured using the Quality of Life Questionnaire Core 30 Items (QLQC30) fatigue and physical functioning subscales (0–100). For fatigue, values >40 were considered as severely fatigued: For physical functioning, values >83 were considered as good physical functioning.

Due to the small number of individuals who had an early retirement or benefited from a disability status between T2 and T3, we were unable to perform any regression analysis. However, results of the analysis grouping having any period of unemployment, retirement or disability were similar to those in Table 3 (Table S2).

## 4 DISCUSSION

In this study, we assessed the effect sociodemographic, clinical, health and work‐related factors had on SRTW among women who were working 2 years after diagnosis. More specifically, we looked at the factors associated with having any sick leave and/or being unemployed at any moment between 2 and 3 years after diagnosis. Although the majority of BCS continuously worked between 2 and 3 years after diagnosis, 23% did not. Understanding the characteristics associated with working discontinuously after having returned to work is essential to help BCS retain their jobs and to avoid long periods of sick leave. We identified that SRTW was associated with health status, clinical, life priorities, sociodemographic and work‐related factors.

Before discussing the results, we would like to address methodological issues discussed. The study population derives from the CANTO cohort, a large cohort of BCS including prospectively collected detailed information on clinical, health, sociodemographic and work‐related factors.^19^ Levels of fatigue, physical functioning and distress were measured using patient‐reported outcomes; however, all work and sociodemographic characteristics were self‐reported. Although perceived discrimination was subjectively measured, the definition of perceived discrimination used in this study mimics that used in previous studies, making results comparable.^12^, ^13^

Furthermore, in our analyses, we excluded self‐employed survivors, whose trajectories can differ from employees.^24^ In addition, we only included BCS, a more homogeneous group compared to most of the literature that investigates all cancers. The large sample size and the diversity of information collected enabled us to study the sub‐population of BCS who had already returned to work and the factors associated with SRTW in this population. Although a lack of statistical power prevented further investigation into retirement and disability, our analyses suggested different profiles exist for those on sick leave on one hand, and those unemployed on the other. Health status is a strong determinant for being on sick leave which suggests that some BCS may have returned to work too early; some people may return to work but are unable to continue working as before.^25^ However, information related to work ability is not collected in CANTO. In contrast, unemployment was not associated to health‐related factors but to characteristics related to work, such as work precariousness.

Second, in line with existing literature, our results highlight the impact of clinical and health factors on sick leave.^6^, ^9^ These results show the importance of the management of sequelae and the need for continuous research on the diminution of severe effects of cancer treatment. Different rehabilitation and behavioural programmes (e.g. physical exercise) exist to help better cope with sequelae, such as chemotherapy‐induced fatigue.^26^ In this regard, workplace accommodations have been designed to facilitate reintegration of cancer survivors on their RTW. Indeed, several studies have suggested their effectiveness in helping survivors return to work.^14^, ^27^ In our study, we found that while workplace accommodations have a protective effect on unemployment, they were unsuccessful at avoiding sick leave. These counterintuitive results are likely to be due to the effect of employers and the dynamic nature of RTW. On the one hand, employers that accommodate to the needs of BCS may be less likely to terminate their contract. Similarly, due to employers' predisposition, employees from companies accommodating to their needs may experience a greater sense of security, feeling that sick leave will not have adverse effects on their professional standing. On the other hand, accommodations and other instruments used to help BCS in their workforce reintegration need to be tailored to their needs, which may change over time.^14^ Based on the evidence, we recommend a joint effort between practitioners, employers and survivors to enhance communication and ensure tailored guidance and rehabilitation programmes are implemented to reduce recurrent sick leaves.

Third, in our population, work characteristics and instability of contracts (e.g. part‐time, fixed‐term contracts) have an important effect on SRTW, especially on unemployment. The effect of types of contracts on job retention may not be exclusive to the cancer population, but such diagnoses may aggravate the precarity effects. Indeed, cancer survivors are more likely to report discriminatory behaviours from employers compared to individuals with other types of disability.^28^ Interestingly, while working for a small company is a protective factors against employer discrimination,^29^ it increases the odds of being unemployed 3 years after diagnosis. This suggests that, despite having a closer employer/employee relationship, smaller companies lack resources to adapt to the needs of BCS. This should be accounted for when designing programmes to help BCS return to work and retain their jobs.

Fourth, our results suggest that age was negatively associated with both outcomes. This may be due to a selection in the >50 year old BCS; BCS in this age group who returned to work before T2 may have returned due to financial constraints or administrative requirement (closer to retirement age).

Finally, changes in how BCS value work is a determinant of RTW after BC.^30^, ^31^ On one hand, work can give a sense of normality to cancer survivors^4^; however, cancer diagnosis is a traumatising life event that can make the person affected reconsider life priorities.^32^ We found that BCS whose professional life was as or more important than their private life are less likely to have sick leave than those for whom their private life is more important. It could be that BCS for whom their private life is more important than their professional life are more likely to take time for themselves. Because this can also be affected by the financial circumstances of the household, it would be interesting if future studies with a larger group of BCS exiting the workforce focus on the interaction between these two factors. In our study, we were unable to further study the effect of those factors on early retirement and disability and this should also be further studied. Finally, future studies should also endeavour to incorporate registry data, which can have a more detailed information on sick leave.

## 5 CONCLUSION

Literature shows cancer survivors have more frequent sick leaves and worse job retention than the general population.^5^, ^10^ The proportion of cancer survivors working peaks at 3–4 years after diagnosis and then decreases again.^5^, ^33^ While most studies have studied RTW,^5^, ^6^, ^7^, ^9^, ^16^ our study investigates job trajectories after BC in more detail and adds to the literature by documenting SRTW among BCS. We found that although most BCS continue to work after RTW, a non‐negligible number of BCS stop working, even temporally, and that factors associated with RTW may not explain SRTW. Indeed, policymakers, companies and practitioners should avoid defining RTW as the end point, but rather as a dynamic process. This dynamicity should be present when designing and implementing measures for SRTW, such as workplace accommodations. Our findings also suggest that there is a need to tailor rehabilitation programmes depending on the profile of the BCS, considering their changing needs over time during the recovery process and the characteristics of companies they work in. If BCS are unable to work continuously with recurrent sick leaves, early retirement and loss of employment, this can have negative public health and economic impact on both the individuals and the society.

## AUTHOR CONTRIBUTIONS

**Garazi Ruiz De Azua:** Conceptualization (equal); data curation (equal); formal analysis (equal); funding acquisition (equal); investigation (equal); methodology (equal); resources (equal); validation (equal); visualization (equal); writing – original draft (equal); writing – review and editing (equal). **Isabelle Kousignian:** Methodology (equal); resources (equal); writing – review and editing (equal). **Ines Vaz‐Luis:** Data curation (equal); resources (equal); writing – review and editing (equal). **Antonio Di Meglio:** Data curation (equal); resources (equal); writing – review and editing (equal). **Elsa Caumette:** Data curation (equal); resources (equal); writing – review and editing (equal). **Julie Havas:** Data curation (equal); resources (equal); writing – review and editing (equal). **Elise Martin:** Resources (equal); writing – review and editing (equal). **Anne‐Laure Martin:** Project administration (equal); resources (equal); writing – review and editing (equal). **Ophelie Querel:** Project administration (equal); resources (equal); writing – review and editing (equal). **Laurence Vanlemmens:** Resources (equal); writing – review and editing (equal). **Barbara Pistilli:** Resources (equal); writing – review and editing (equal). **Charles Coutant:** Resources (equal); writing – review and editing (equal). **Paul Henri Cottu:** Resources (equal); writing – review and editing (equal). **Asma Dhaini Merimeche:** Resources (equal); writing – review and editing (equal). **Florence Lerebours:** Resources (equal); writing – review and editing (equal). **Olivier Tredan:** Resources (equal); writing – review and editing (equal). **Christelle Jouannaud:** Resources (equal); writing – review and editing (equal). **Christelle Levy:** Resources (equal); writing – review and editing (equal). **Agnes Dumas:** Data curation (equal); resources (equal); writing – review and editing (equal). **Gwenn Menvielle:** Conceptualization (equal); data curation (equal); funding acquisition (equal); investigation (equal); methodology (equal); resources (equal); supervision (equal); validation (equal); visualization (equal); writing – original draft (equal); writing – review and editing (equal).

## FUNDING INFORMATION

The CANTO study is supported by the French Government under the ‘Investment for the Future’ programme managed by the National Research Agency (ANR), grant n°ANR‐10‐COHO‐0004 and Fondation ARC pour la Recherche sur le Cancer (number PGA1 RF20170205420). Garazi Ruiz de Azua's research was funded by Ligue Contre le Cancer.

## CONFLICT OF INTEREST STATEMENT

Christelle Jouannaud: Honoraria (Pfizer, Daiichi Sankyo/Astra Zeneca).

## ETHICS APPROVAL STATEMENT

The study was approved by the national regulatory authorities and ethics committee (ID‐RCB: 2011‐A01095‐36, 312 11–039). CANcer TOxicities is a trial research involving the humans benefiting from authorisation from the French National Agency for the Safety of Medicines and Health Products obtained on 14 September 2011 (number B111158‐20) and from the Ile‐de‐France VII Committee for the Protection of Individuals obtained on 14 October 2011 (number 11‐039).

## INFORMED CONSENT STATEMENT

Informed consent was obtained from all subjects involved in the study.

## CLINICAL TRIAL REGISTRATION

NCT01993498.

## Supporting information


Table S1–S2

## Data Availability

CANTO data are not publicly available and can be obtained from UNICANCER.
