# Supplementary material for: Sustainable return to work among breast cancer survivors
Source: Cancer Med. 2023 Aug 21;12(18):19091–101. doi: 10.1002/cam4.6467 (PMC10557874; doi:10.1002/cam4.6467)
Supplement: Supplementary file 1 — Table S1–S2 [file CAM4-12-19091-s001.docx]

**Table S1. Distribution (before imputation) of complete study sample and by having worked continuously, any sick leave or any unemployment period between T2 and T3*.**

|  | **All**  **N=1811** (100%)** | **Worked continuously**  **N=1395 (100%)** | **Any sick leave N=276 (100%)** | **Any unemployment N=151 (100%)** |
| --- | --- | --- | --- | --- |
| **Sociodemographic characteristics at diagnosis** |  |  |  |  |
| Age >=50 *(ref. <50)* | 704 (39) | 567 (47) | 81 (29) | 40 (30) |
| *NA* | 0 | 0 | 0 | 0 |
| **Clinical factors at diagnosis** |  |  |  |  |
| Stage at dx *(ref. I)* | 848 (47) | 672 (49) | 103 (38) | 69 (51) |
| *II* | 777 (43) | 598 (43) | 118 (43) | 59 (44) |
| *III* | 173 (10) | 114 (8) | 53 (19) | 7 (5) |
| *NA* | 13 | 11 | 2 | 0 |
| Tumour subtype *(ref. HR+/HER2-)* | 1342 (75) | 1020 (73) | 208 (76) | 15 (11) |
| *HR+/HER2+* | 220 (12) | 180 (13) | 28 (10) | 3 (2) |
| *HR-/HER2+* | 69 (4) | 48 (4) | 18 (7) | 107 (80) |
| *HR-/HER2-* | 170 (9) | 140 (10) | 20 (7) | 8 (6) |
| *NA* | 10 | 7 | 2 | 2 |
| **Health status two years after diagnosis** |  |  |  |  |
| Good physical functioning^c^ *(ref. Bad)* | 1379 (77) | 1086 (79) | 187 (68) | 101 (76) |
| *NA* | 25 | 32 | 1 | 2 |
| Severely fatigued^c^ *(ref. No)* | 655 (37) | 468 (34) | 135 (49) | 51 (38) |
| *NA* | 28 | 24 | 1 | 2 |
| Distress^c^ *(ref. No distress)* | 1114 (63) | 877 (65) | 154 (56) | 80 (62) |
| *Distress: Borderline* | 440 (25) | 336 (25) | 68 (25) | 31 (24) |
| *Distress: Case* | 209 (12) | 141 (10) | 52 (19) | 19 (15) |
| *NA* | 48 | 41 | 2 | 5 |
| **Sociodemographic characteristics two years after diagnosis** |  |  |  |  |
| Household income *(ref. <2500€)* | 425 (25) | 313 (23) | 68 (26) | 42 (34) |
| *2500€-5000€* | 966 (56) | 753 (56) | 141 (56) | 69 (56) |
| *>5000€* | 329 (19) | 267 (20) | 47 (18) | 13 (10) |
| *NA* | 91 | 62 | 16 | 11 |
| Lives with partner *(ref. No)* | 1379 (77) | 1067 (77) | 206 (75) | 100 (74) |
| *NA* | 19 | 16 | 2 | 0 |
| Has economically dependent children *(ref. No)* | 1116 (64) | 861 (65) | 179 (67) | 79 (60) |
| *NA* | 78 | 62 | 8 | 4 |
| **Work and workplace factors two years after diagnosis** |  |  |  |  |
| Had workplace accommodations *(ref. No)* | 1041 (62) | 787 (61) | 204 (79) | 51 (44) |
| *NA* | 134 | 99 | 17 | 19 |
| Reported perceived discrimination *(ref. No)* | 457 (26) | 331 (24) | 98 (37) | 31 (26) |
| *NA* | 52 | 30 | 8 | 14 |
| Size of company *(ref. Large)* | 745 (44) | 589 (45) | 130 (50) | 21 (18) |
| *Medium* | 368 (22) | 290 (22) | 55 (51) | 25 (22) |
| *Small* | 580 (34) | 435 (33) | 75 (79) | 69 (60) |
| *NA* | 118 | 81 | 16 | 20 |
| Has a fixed-term contract *(ref. No)* | 247 (14) | 161 (12) | 30 (11) | 54 (44) |
| *NA* | 71 | 46 | 11 | 12 |
| Works part time *(ref. No)* | 648 (37) | 459 (34) | 137 (51) | 52 (43) |
| *NA* | 62 | 37 | 9 | 15 |
| Works in the public sector *(ref. No)* | 781 (45) | 626 (46) | 121 (46) | 29 (24) |
| *NA* | 72 | 41 | 13 | 13 |
| Returned to work because of fear of job loss *(ref. No)* | 338 (19) | 245 (18) | 63 (24) | 33 (26) |
| *NA* | 47 | 30 | 10 | 7 |
| Professional life is as or more important than private life *(ref. No)* | 763 (43) | 619 (45) | 90 (34) | 55 (45) |
| *NA* | 47 | 24 | 10 | 12 |

* T2, T3: two and three years after diagnosis. **1811 includes those on working continuously between T2 and T3, and those with any sick leave, unemployment, early retirement or disability between T2 and T3. Please note that some BCS had several events between T2 and T3.

**Table S2. Association between being unemployed, on early retirement or benefiting from a disability status between T2 and T3 and sociodemographic, clinical, health and work-related factors compared to having worked continuously between T2 and T3 (logistic regression) (N=1546).**

|  | **Univariable models^a^** | | | | **Multivariable models^a^** | |
| --- | --- | --- | --- | --- | --- | --- |
|  | **OR^b^** | | | **[95%CI]** | **ORa^b^** | **[95%CI]** |
| **Sociodemographic characteristics at dx^b^** |  | | |  |  |  |
| Age >=50 *(ref. <50)* | 0.77 | [0.54;1.09] | | | 0.58 | [0.38;0.89] |
| **Clinical factors at dx^b^** |  | |  | |  |  |
| Stage at dx *(ref. I)* |  | |  | |  |  |
| *II* | 1.04 | | [0.73;1.47] | | 1.07 | [0.73;1.58] |
| *III* | 0.63 | | [0.29;1.33] | | 0.73 | [0.32;1.66] |
| Tumour subtype *(ref. HR+/HER2-)* |  | |  | |  |  |
| *HR+/HER2+* | 0.83 | | [0.49;1.41] | | 0.97 | [0.54;1.75] |
| *HR-/HER2+* | 0.79 | | [0.28;2.18] | | 0.96 | [0.31;2.92] |
| *HR-/HER2-* | 0.57 | | [0.28;1.14] | | 0.55 | [0.26;1.16] |
| **Health status two years after dx^b^** |  | |  | |  |  |
| Good physical functioning^c^ *(ref. Bad)* | 0.75 | | [0.51;1.10] | | 0.77 | [0.47;1.25] |
| Severely fatigued^c^ *(ref. No)* | 1.27 | | [0.90;1.80] | | 1.08 | [0.70;1.66] |
| Distress^c^ *(ref. No distress)* |  | |  | |  |  |
| *Distress: Borderline* | 0.96 | | [0.63;1.46] | | 0.91 | [0.57;1.47] |
| *Distress: Case* | 1.54 | | [0.94;2.53] | | 1.35 | [0.73;2.47] |
| **Sociodemographic characteristics two years after dx^b^** |  | |  | |  |  |
| Household income *(ref. <2500€)* |  | |  | |  |  |
| *2500€-5000€* | 0.75 | | [0.51;1.10] | | 1.08 | [0.66;1.79] |
| *>5000€* | 0.43 | | [0.24;0.78] | | 0.86 | [0.42;1.78] |
| Lives with partner *(ref. No)* | 0.87 | | [0.59;1.28] | | 0.74 | [0.44;1.24] |
| Has economically dependent children *(ref. No)* | 0.77 | | [0.54;1.09] | | 0.70 | [0.45;1.07] |
| **Work and workplace factors two years after dx^b^** |  | |  | |  |  |
| Had workplace accommodations *(ref. No)* | 0.59 | | [0.41;0.83] | | 0.55 | [0.36;0.86] |
| Reported perceived discrimination *(ref. No)* | 1.11 | | [0.75;1.65] | | 1.32 | [0.82;2.12] |
| Size of company *(ref. Large)* |  | |  | |  |  |
| *Medium* | 1.97 | | [1.14;3.41] | | 1.63 | [0.92;2.89] |
| *Small* | 3.61 | | [2.31;5.63] | | 2.78 | [1.68;4.61] |
| Has a fixed-term contract *(ref. No)* | 5.24 | | [3.60;7.64] | | 6.45 | [4.18;9.97] |
| Works part time *(ref. No)* | 1.71 | | [1.19;2.46] | | 1.79 | [1.15;2.76] |
| Works in the public sector *(ref. No)* | 0.47 | | [0.32;0.70] | | 0.40 | [0.26;0.64] |
| Returned to work because of fear of job loss *(ref. No)* | 1.56 | | [1.05;2.32] | | 1.36 | [0.86;2.13] |
| Professional life is as or more important than private life *(ref. No)* | 0.87 | | [0.61;1.24] | | 0.79 | [0.53;1.18] |

^a^ Reference category is having worked continuously between T2 and T3. ^b^dx= diagnosis; ORa= adjusted adds ratio; T2, T3: two and three years after diagnosis. ^b^ Severe fatigue and physical functioning were measured using the Quality of Life Questionnaire Core 30 Items (QLQC30) fatigue and physical functioning subscales (0-100). For fatigue values >40 were considered as severely fatigued: for physical functioning values >83 were considered as good physical functioning.

Distress was evaluated using the general subscale of HADS which combines the anxiety and depression specific subscales of the Hospital Anxiety and Depression Scale (HADS). 0-12: normal, 13-18: borderline; 19-42: case.
